# Performance Assessment of a Hybrid Solar-Driven Photocatalysis–Membrane Distillation Process for the Removal of Ketoprofen from Seawater

**DOI:** 10.3390/membranes16090280

**Published:** 2026-08-22

**Authors:** Kacper Szymański, Alba Ruiz-Aguirre, Aleksandra Piątkowska, Sylwia Mozia, Guillermo Zaragoza

**Affiliations:** 1 Department of Inorganic Chemical Technology and Environment Engineering, Faculty of Chemical Technology and Engineering, West Pomeranian University of Technology in Szczecin, Pułaskiego 10, 70-322 Szczecin, Poland; 2 Center for Advanced Materials and Manufacturing Process Engineering (CAMMPE), Faculty of Chemical Technology and Engineering, West Pomeranian University of Technology in Szczecin, al. Piastów 42, 71-065 Szczecin, Poland; 3 CIEMAT-Plataforma Solar de Almeria, Ctra. de Senés s/n, 04200 Tabernas, Spain; 4 Centro Mixto CIESOL, Universidad de Almería, Ctra. Sacramento s/n, 04120 Almeria, Spain

**Keywords:** solar-driven photocatalysis, air gap membrane distillation, pharmaceutical, ketoprofen, seawater

## Abstract

In the present research, the application of a photocatalytic reactor operated under simulated solar-light-assisted air gap membrane distillation (AGMD) is proposed to remove ketoprofen from seawater. TiO_2_ at a concentration of 1 g/L, containing sulfur, was applied as a photocatalyst. The AGMD process was carried out under a feed temperature of 60–80 °C and a membrane area of 131 cm^2^ during long-term operation. Simulated solar light was applied as an irradiance source. At the first stage of the process, the feed was concentrated for 73 h, and after that, the solution of seawater spiked with ketoprofen was photocatalytically treated for 96 h. Based on the experiments, it was found that 51% of ketoprofen was removed after the solar-driven photocatalysis process. Pure distillate without salts (conductivity below 2 µS/cm) and ketoprofen were obtained after 73 h. The performance of the membrane exhibited ca. two times higher permeate flux at an operation temperature of 80 °C in comparison with 60 °C, i.e., 24.7 L/h·m^2^ and 47.3 L/h·m^2^, respectively. Despite the presence of small deposits on the membrane surface, no membrane wetting was observed. The concentration of ketoprofen in the concentrates during the AGMD process and solar-driven photocatalysis can remove this pharmaceutical even from matrices enriched with salts (high AGMD concentrate), with good efficiency.

## 1. Introduction

Most wastewater treatment plants are not capable of efficiently removing contaminants of emerging concern (CECs), such as pharmaceuticals, resulting in the presence of these compounds in aquatic ecosystems. This can lead to problems such as toxicity and the development of antibiotic resistance. On 1 January 2025, the European Directive 2024/3019 came into force, requiring all wastewater treatment plants (WWTPs) with a load equal to or greater than 150,000 population equivalents, as well as certain selected plants, to implement quaternary treatment processes for the removal of micropollutants, including pharmaceuticals, with a minimum efficiency of 80% [1]. One of the promising solutions is advanced oxidation processes (AOPs) and membrane-based processes. The former can effectively remove contaminants, but challenges remain. Although AOPs can achieve high removal rates, they may not always lead to complete mineralization, resulting in the formation of transformation products that can be more persistent or even more toxic than the original compound. This makes post-treatment necessary [2]. Membrane filtration technologies are capable of achieving high levels of separation and concentration, but some of them, such as ultrafiltration (UF) and microfiltration (MF), do not provide a complete barrier against contaminants. However, nanofiltration (NF), forward osmosis (FO), and reverse osmosis (RO) are more efficient in the rejection of pharmaceuticals, reaching high concentration rates. Thus, these membrane technologies have the potential to be used for preconcentrating wastewater and reducing its volume before applying another treatment to eliminate these contaminants, such as advanced oxidation processes (AOPs). So, their combination with membrane technologies can offer several advantages. AOPs are generally governed by pseudo-first-order kinetics with respect to the contaminants. This means that the degradation rate depends linearly on the concentration of the contaminant when the oxidant is in excess. Therefore, if the contaminant concentration is increased, the initial reaction rate also increases—up to a certain limit. By concentrating the contaminant, the rate at which its concentration decreases is accelerated, and the residence time required to reach a certain level of degradation is reduced. Also, the radicals are more likely to react with the target contaminant, improving oxidation efficiency [3]. However, this type of hybrid approach has been scarcely explored in the current scientific literature, especially for addressing pharmaceuticals [4]. Membrane distillation (MD) is a thermal separation technology in which only vapor molecules are able to pass through a porous hydrophobic membrane [5]. The feed solution comes into direct contact with the membrane’s surface, but it does not penetrate the membrane due to its hydrophobic properties. The driving force for the process is the vapor pressure gradient across the membrane, which is typically created by heating the feed solution on one side of the membrane, raising its vapor pressure and thereby creating a gradient across the membrane [5]. MD presents several advantages that make it attractive and competitive for water treatment: (i) MD can achieve nearly 100% rejection of non-volatile contaminants, including salts, heavy metals, pharmaceuticals, etc.; (ii) it is thermally driven, so there is no need for high-pressure pumps, and there is lower mechanical stress and less membrane fouling; (iii) MD is more tolerant to fouling and scaling, especially for high-salinity or organic-rich feedwaters; (iv) it can operate at higher recovery rates for saline or complex streams; (v) it can treat highly concentrated and toxic brines [5]. Qoriati et al. [6] successfully employed air gap membrane distillation, which is one of the variations of the MD process for nutrient and water recovery from marine culture wastewater. As an optimal parameter for process efficiency following temperature, the pH value was highlighted [6]. Advanced oxidation processes (AOPs) have proven to be highly effective in breaking down persistent emerging contaminants (ECs). However, their large-scale application faces significant obstacles due, among other factors, to high electricity demands (use of UV lamps). Solar radiation is a renewable and abundant energy source that can be easily integrated into photocatalytic processes, making them more environmentally sustainable. Considerable efforts have been dedicated to investigating the feasibility of operating these processes under solar irradiation. In recent years, the performance of solar photocatalysis for the removal of micro-pollutants in municipal wastewater treatment plant (MWTP) effluents under various operational conditions has been widely studied [7,8,9]. However, treatment rates are generally slow due to the initially low concentrations of contaminants and the pseudo-first-order kinetics involved (r = kapC) [10]. To enhance process efficiency, one proposed strategy is to increase the initial concentration of the contaminant (C_0_), which is applied in the present investigation. The potential to achieve this by integrating AOPs with membrane processes has drawn increasing interest in recent years, particularly for pharmaceutical removal. This is especially important in the case of ketoprofen, for which concentrations vary across different seawaters. The need for its removal is significant, since the concentration of contaminants in retentates can be substantially higher than in the raw effluents from municipal wastewater treatment plants (MWTPs). Recently, our research group proposed a new hybrid SPMR-DCMD system with a labyrinth flow photoreactor to treat various feed water matrices contaminated with ketoprofen [11,12]. It was revealed that feed composition was mainly responsible for the efficiency of photodegradation of organic contaminants and affected distillate quality. Nevertheless, this system employed a hollow fiber capillary membrane for direct-contact membrane distillation and UV- or simulated solar-driven photocatalysis in one unit. The present work revealed the impact of highly salted feed water on ketoprofen removal in the combined air gap membrane distillation (AGMD)–photocatalysis system. It should also be emphasized that the presented configuration was investigated under simulated solar light. Such a system simulates ketoprofen removal from seawater at pilot-plant scale. Apart from our works [11,12], there are no reports in the literature on the photocatalytic decomposition of ketoprofen, especially using a photocatalytic reactor coupled with an air gap membrane distillation process, under solar light. The removal of three various pharmaceuticals (ibuprofen, naproxen, and diclofenac) in a PMR utilizing direct contact membrane distillation (DCMD) was previously investigated by our research group [13]; however, those studies applied UV light photocatalysis. The effectiveness of pharmaceutical removal was strongly correlated with feed matrix components [13]. Moreover, the presented system differs in that, in this case, the membrane distillation process was carried out first, followed by the photocatalytic process after concentration. In contrast, in previous studies, membrane distillation (MD) and photocatalysis occurred simultaneously, which may lead to the transfer of volatile degradation compounds into the distillate and cause membrane fouling and partial wetting by photocatalyst particles [12]. The literature also describes another approach, where the photocatalyst is deposited on the membrane or incorporated into its structure [14]. However, such a solution may lead to polymer degradation and damage to the membrane [15]. The studies based on PMR and MD mostly focused on the removal of phenol and dyes for a short period of time [16,17,18,19]. Yatmaz et al. [18] carried out experiments in which the photodegradation of Reactive Red 180 and Reactive Orange 16 in a system equipped with a reactor under UV-A and UV-C light was investigated. Two types of photocatalysts (TiO_2_ and ZnO) were applied. A membrane module with a flat sheet membrane was divided from the photoreactor [18]. Huo et al. [19] studied a hybrid membrane distillation–photocatalysis process using BiOBr as a photocatalyst active under visible light for methyl orange removal. The light source was positioned above the feed tank. A hydrophobic polytetrafluoroethylene flat sheet membrane was applied. The proposed configuration was very effective for the photodecomposition of the dye, and the photocatalyst was completely separated by the membrane without any fouling due to the micrometer size of the particles [19]. The literature reports systems employing modified photocatalysts designed for enhanced visible/solar-light activity in the removal of pharmaceuticals from highly saline solutions. However, these studies were conducted in conjunction with reverse osmosis rather than membrane distillation [20].

Since micropollutants usually occur in complex matrices and the removal of pharmaceuticals in MD is not obvious and depends on the type of pharmaceutical, further research is necessary to evaluate the influence of feed components on the effectiveness of photocatalysis–MD in more realistic reaction media. The cost of implementing solar-driven photocatalysis for commercial use can be greatly reduced by applying solar light. Moreover, utilizing solar light for photodecomposition purposes represents a green, sustainable, and cost-effective solution for regions with a high correlation between water shortage and high solar irradiance, which creates opportunities for obtaining purified water in countries with water scarcity and the possibility of using solar energy. Therefore, the main aim of this work is to evaluate the removal of the pharmaceutical ketoprofen from seawater in a system based on air gap membrane distillation and solar-driven photocatalysis. A key advantage of this approach is the prevention of membrane scaling and mechanical deterioration, which minimizes the likelihood of membrane wetting. Consequently, the risk of ketoprofen and/or volatile organic byproducts generated during ketoprofen degradation, which permeate through the membrane and contaminate distillates, is significantly reduced.

## 2. Materials and Methods

### 2.1. Air Gap Membrane Distillation (AGMD) System

The membrane used in this module was a commercial membrane from General Electric Energy (GE Energy). This membrane consisted of a hydrophobic PTFE active layer laminated onto a PP support structure that provided mechanical strength. The nominal pore size and porosity were 0.2 μm and 85%, respectively. The membrane’s thickness ranged from 0.12 to 0.2 mm, and the liquid entry pressure (LEP) was greater than 3.5 bar. The membrane surface measured 29 × 13 cm; however, the actual effective surface area was 131 cm^2^, which was used for the calculation of the permeate flux.

**Figure 1:**
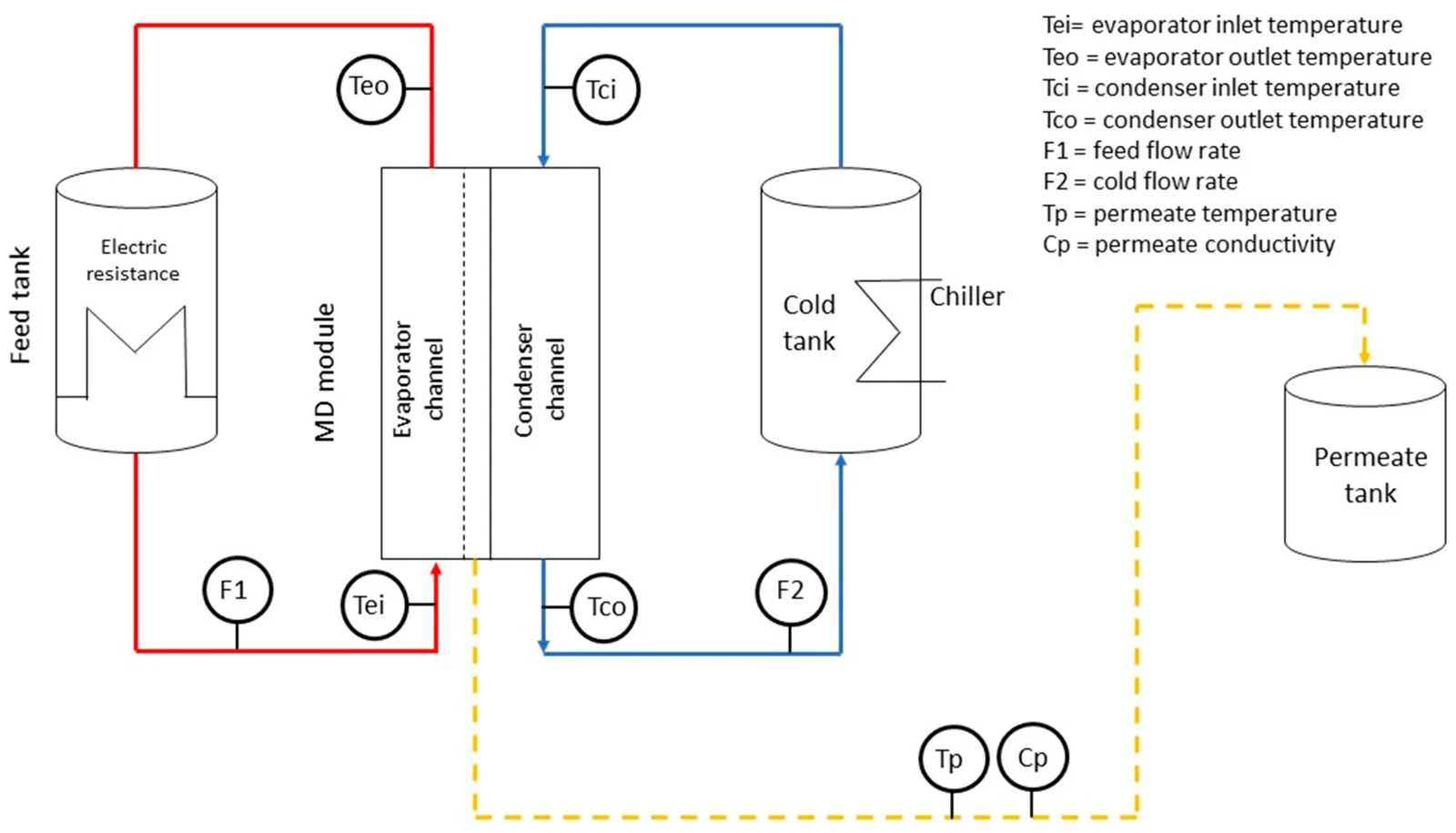
Hydraulic layout of the AGMD system.

The bench-scale MD system was acquired from the company Apria Systems (Guarnizo, Cantabria, Spain). The MD module has a plate-and-frame configuration and was operated in an AGMD configuration. This was integrated into a system consisting of two hydraulically separated loops: (i) one for the hot feed solution and (ii) another for the cooling solution (Figure 1). The feed solution was stored in an 80 L thermally insulated stainless-steel tank (MECALIA DPI/E-80, 316L, MECALIA, Asteasu, Spain), with a design pressure of 8 bar and a maximum temperature of 90 °C. Heating was provided via a 3 kW electric-resistance on–off controller with an external PTC temperature probe (SONDER ECS 57, Sonder Regulacion S.A., Rubi, Spain). The thermostat activated when the measured temperature deviated by ≥0.3 °C from the setpoint. Until the feed reached the target temperature, it was recirculated through the hot loop, bypassing the MD module via a stainless-steel feed pump (GRUNDFOS CM1-2G, Grundfos, Bjerringbro, Denmark; max flow: 2.5 m^3^/h; max pressure: 1.77 bar). Once the set temperature was reached, the feed entered the MD module through the bottom of the evaporation channel and exited from the top, returning to the tank.

The cooling loop used demineralized water stored in another 80 L insulated stainless-steel tank (same characteristic of the feed tank), which was cooled by an internal coil connected to a POLYSCIENCE 6200 chiller (PolyScience, Niles, IL, USA) (800 W at 20 °C; max flow: 11.7 L/min; max pressure: 0.66 bar) and controlled via an external PT100 probe (POLYSCIENCE 6250, PolyScience, Niles, IL, USA). Similarly to the feed, cooling water was recirculated in a closed loop using the same model of pump until it reached the desired temperature, after which it was circulated through the MD module, entering the condensation channel from the top and exiting at the bottom to return to the tank and thereby creating a countercurrent flow with respect to the evaporator channel.

The produced permeate was collected in a separate tank. Temperatures at the inlets and outlets of both evaporation and condensation channels were monitored using four PT100 sensors (PCE WTR120, PCE Instruments, Meschede, Germany). The feed flow rate was controlled with a magnetic inductive stainless-steel rotameter (Tecfluid M21 HRA/inox, Tecfluid S.A., Sant Just Desvern, Spain, range: 60–630 L/h), and cooling flow was adjusted with a PP rotameter (Tecfluid PS31/PVC, Tecfluid S.A., Sant Just Desvern, Spain, range: 60–630 L/h). Four pressure gauges (0–2.5 bar) were installed at the inlet and outlet of both channels. Additionally, two sampling valves were located at the outlets of the evaporation and condensation channels. For safety, two pressure relief valves set to 3 bar were installed in each hydraulic line. The system was equipped with a Memograph M RSG40 (Endress+Hauser, Reinach, Switzerland) for automatic recording of the four temperatures and the feed flow rate.

### 2.2. AGMD Experimental Plan

Heat experiments with the simulated seawater (35 g/L of NaCl) spiked with ketoprofen (50 µg/L) were conducted over a period of 73 h. Ketoprofen (C_16_H_14_O_3_) was purchased from Sigma-Aldrich, St. Louis, MO, USA. The feed tank was filled with 75 L of the feed water. It was then recirculated in a closed loop while being heated to 60 °C or 80 °C. The cooling tank was filled with demineralized water and recirculated in a closed loop while being cooled down to 20 °C. Once the desired temperatures were reached, the ketoprofen solution was added to the feed tank to achieve an initial concentration of 25 µg/L, and the solution was homogenized by pumping in a closed loop. Then, the first sample was taken from the feed tank (t = 0 min), and the contaminated feed solution began flowing through the evaporation channel of the MD module at a flow rate of 400 L/h. On the other side, cooling water also started circulating through the condensation channel at a flow rate of 400 L/h. Both the feed solution and cooling water were recirculated to their respective tanks throughout the experiment. Feed and permeate samples were collected manually at approximately hourly intervals during the experiment. As the sampling procedure was not automated, measurements were not collected during periods when the experimental team was not present at the PSA, although the system continued operating under continuous conditions.

### 2.3. Photocatalytic Installation Setup

During photocatalytic experiments, a laboratory-obtained sulfur-doped TiO_2_, referred to as the P600 photocatalyst, active under solar light at a loading of 1 g/L, was applied. The detailed synthesis and characteristics of the photocatalyst are published elsewhere [21]. Briefly, crude TiO_2_ obtained from a chemical factory was ground in an agate mortar to obtain a fine powder, which was subsequently calcined at 600 °C for 1 h.

**Figure 2:**
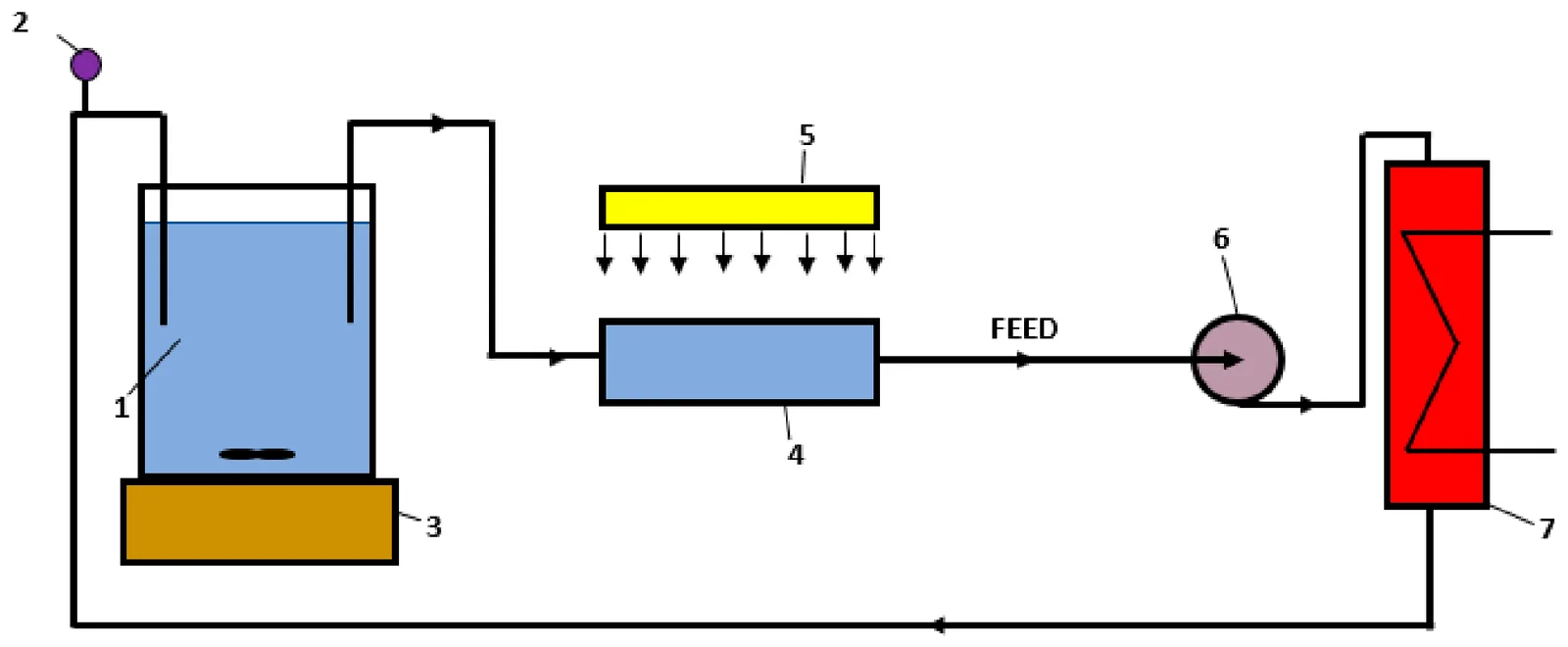
Schematic diagram of the photocatalytic membrane reactor installation setup: 1—feed tank; 2—thermometer; 3—magnetic stirrer; 4—photoreactor; 5—irradiation source; 6—peristaltic pump; 7—heat exchanger.

A schematic diagram of the installation is presented in Figure 2. Simulated solar light was emitted by three bulbs (Daylight Basking Spot, Exo Terra, Montreal, QC, Canada). The irradiation intensity of each bulb was 2000 W/m^2^ in the wavelength range of 300–2800 nm and 0.6 W/m^2^ in the UV-A and UV-B wavelength ranges. The feed at this stage of the research was concentrated via the AGMD process, and it was collected after 73 h (75 µg/L of ketoprofen). The photocatalytic experiment was carried out for 96 h at 25 °C. Before running experiments, the adsorption stage took place for 0.5 h under dark conditions.

### 2.4. Analytical Methods

The concentration of pharmaceuticals was measured using a high-performance liquid chromatograph (HPLC, Shimadzu, Kyoto, Japan). Conductivity and total dissolved solids (TDSs) were analyzed using UltrameterTM 6P (MYRON L COMPANY, Carlsbad, CA, USA). The surface of the membrane samples before and after operation was examined based on laser scanning microscopy (LSM) images. For this purpose, a LEXT OLS5100 microscope (Evident Europe GmbH, Hamburg, Germany) was used. The LSM analysis of as-collected samples was conducted at 50× magnification. The surface roughness parameters (S_a_—i.e., arithmetic mean height calculated as the mean difference in height from the mean plane; S_q_—i.e., squared mean height corresponding to the standard deviation of height distribution) were calculated using the LEXT OLS5100 software from 3 different images. Field emission scanning electron microscopy (SEM, Thermo Scientific Apreo 2, 20 kV, Thermo Fisher Scientific, Eindhoven, The Netherlands) and energy-dispersive spectroscopy (EDS, Thermo Scientific ChemiSEM, Thermo Fisher Scientific, Eindhoven, The Netherlands) were used to observe the morphology of the samples and the distribution of elements on the surface.

## 3. Results

### 3.1. AGMD Processes

**Figure 3:**
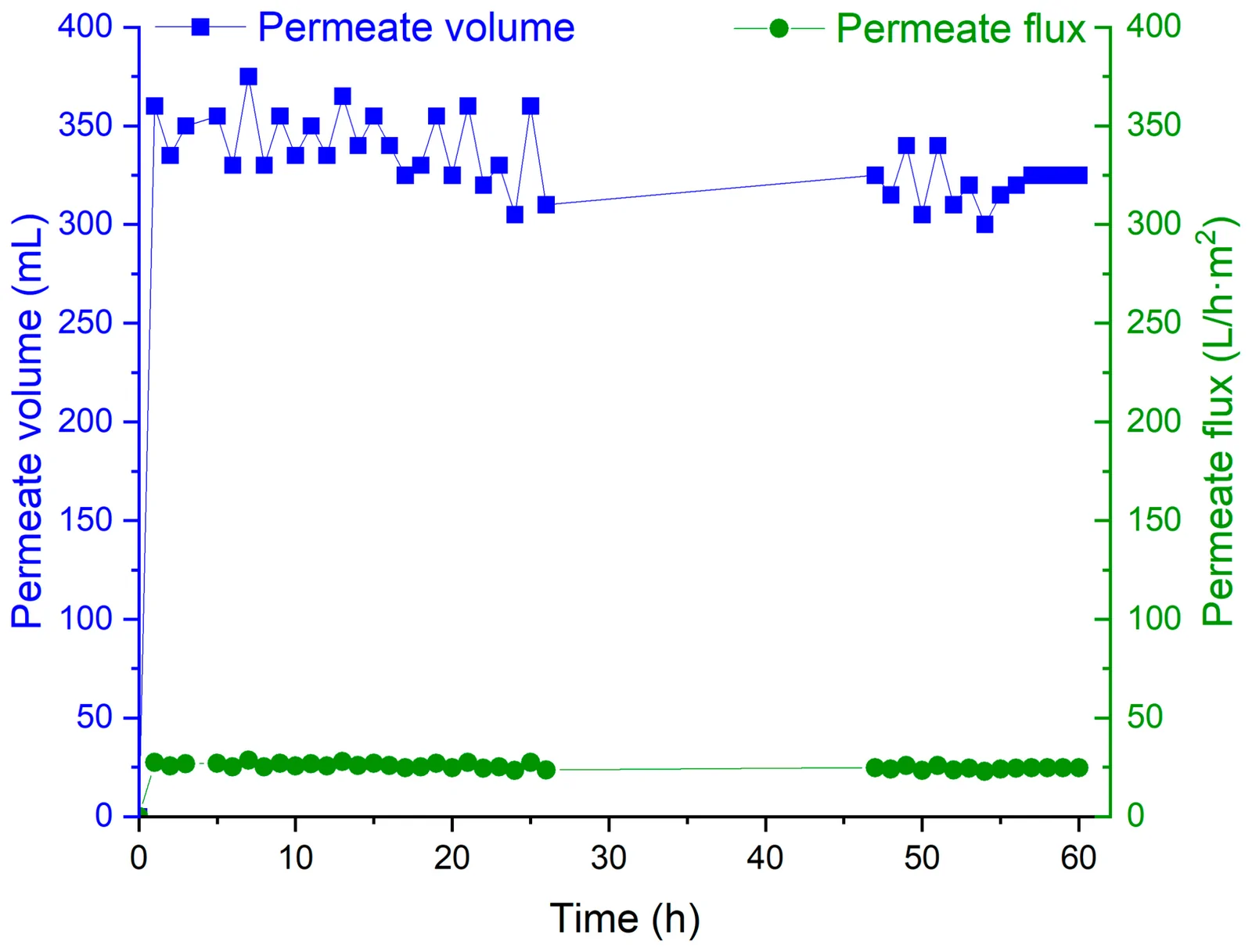
Permeate volume and permeate flux obtained during 60 h of operation at 60 °C. Flow rate: 400 L/h. Measurements were collected manually at approximately hourly intervals. The absence of data points is due to the lack of manual measurements during this period, while the system remained in continuous operation.

**Figure 4:**
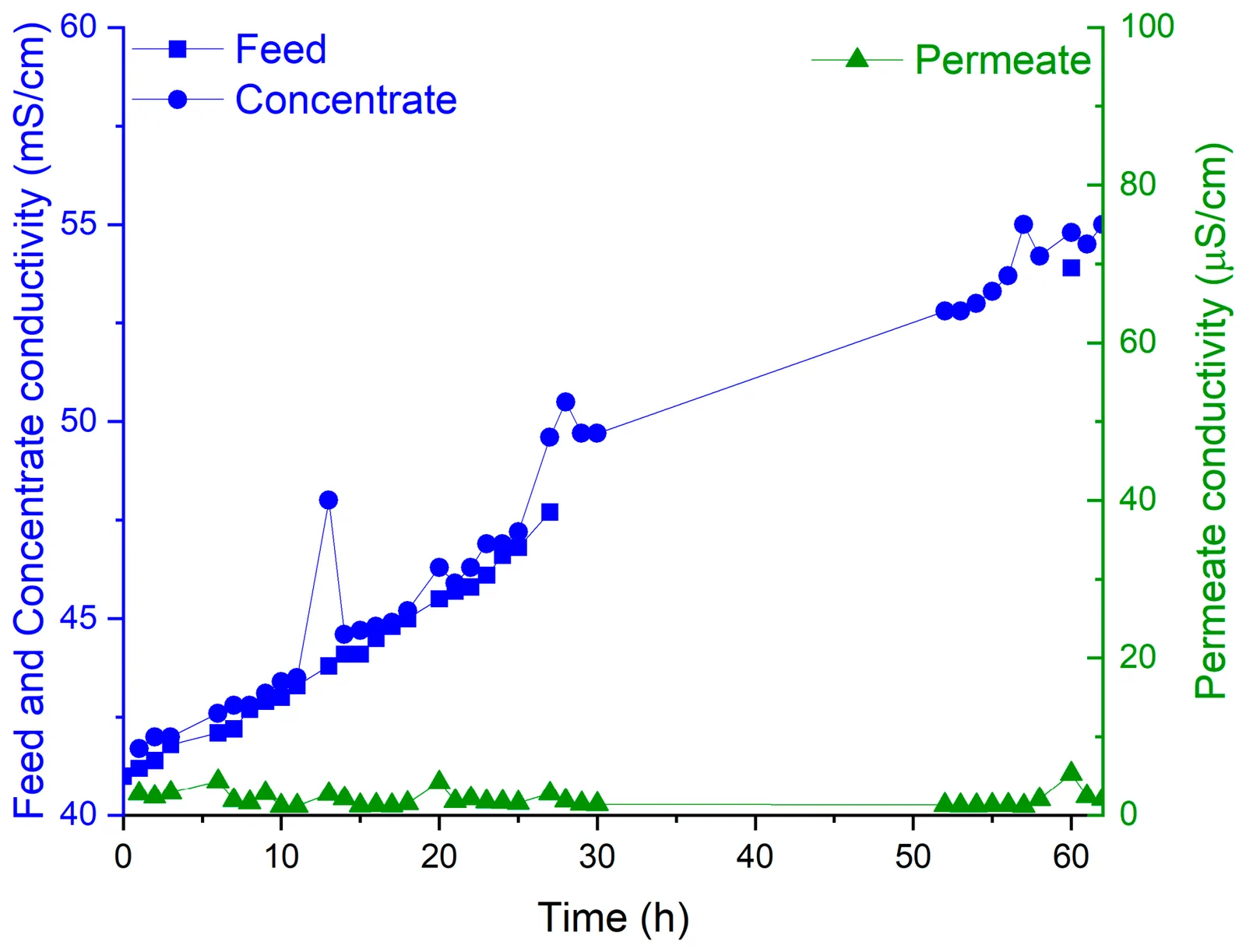
Feed, concentrate, and permeate conductivities obtained during 60 h of operation at 60 °C. Flow rate: 400 L/h. Measurements were collected manually at approximately hourly intervals. The absence of data points is due to the lack of manual measurements during this period, while the system remained in continuous operation.

**Figure 5:**
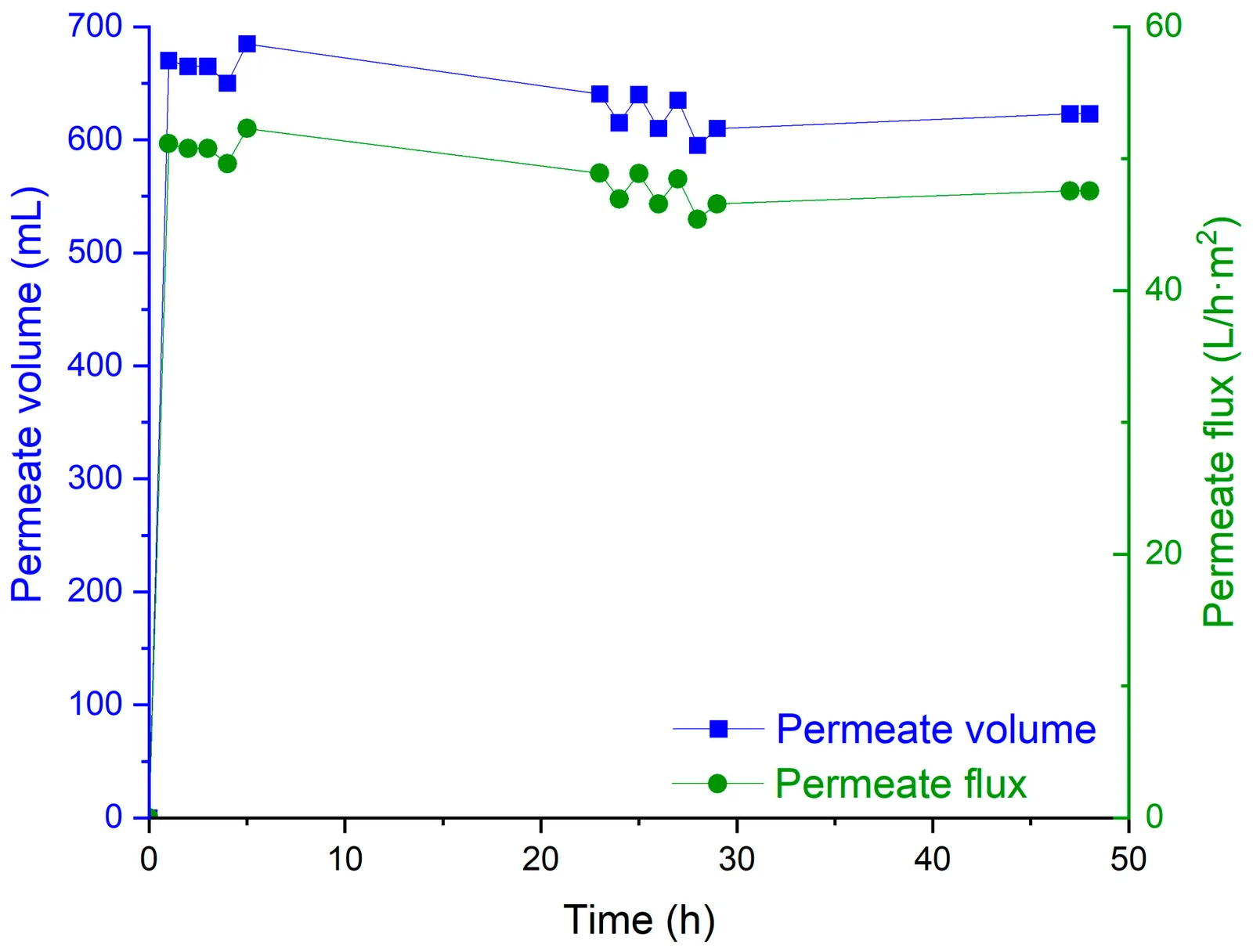
Permeate volume and permeate flux obtained during 48 h of operation at 80 °C. Flow rate: 400 L/h. Measurements were collected manually at approximately hourly intervals. The absence of data points is due to the lack of manual measurements during this period, while the system remained in continuous operation.

**Figure 6:**
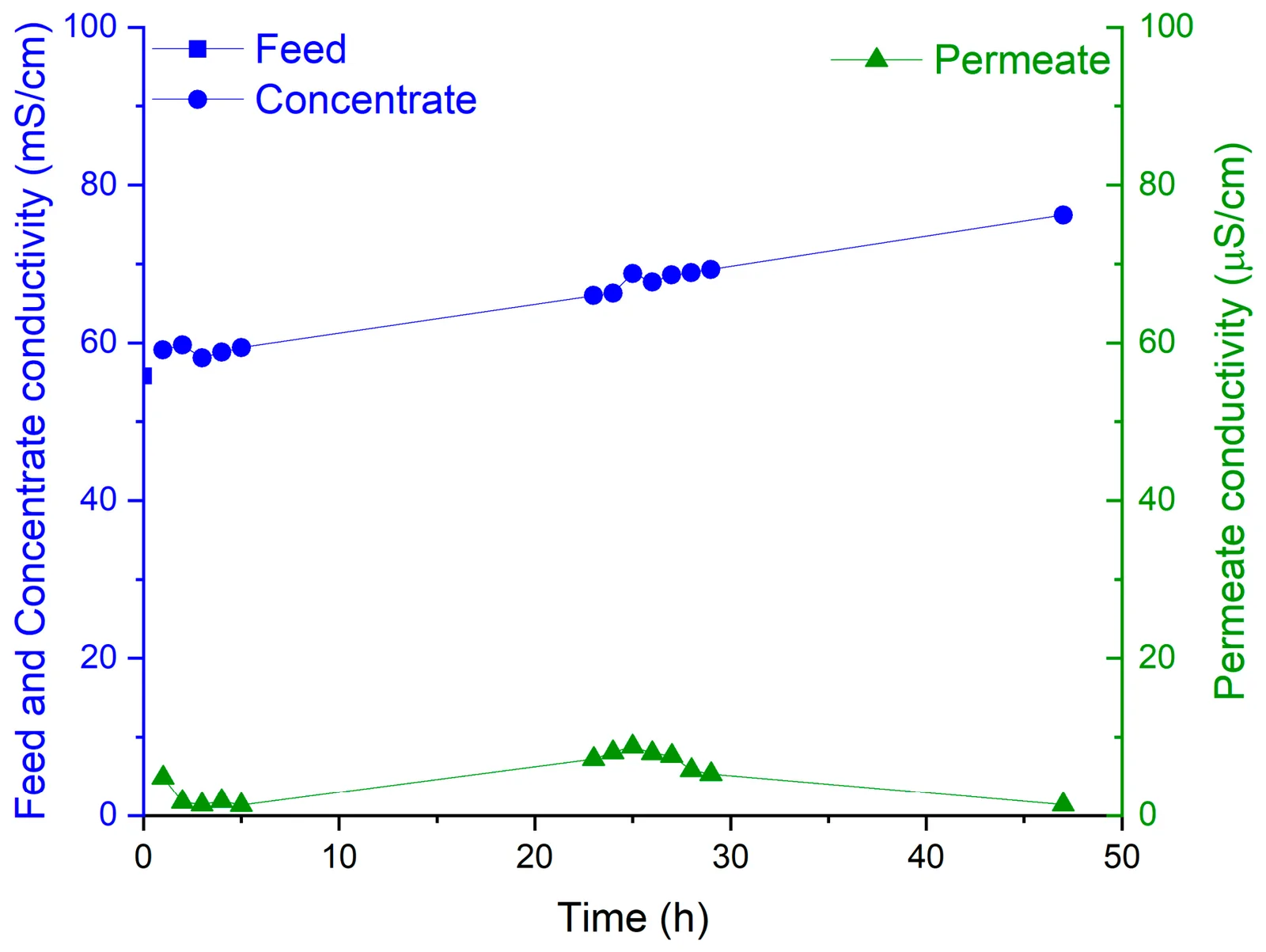
Feed, concentrate, and permeate conductivities obtained during 48 h of operation at 80 °C. Flow rate: 400 L/h. Measurements were collected manually at approximately hourly intervals. The absence of data points is due to the lack of manual measurements during this period, while the system remained in continuous operation.

MD membranes are hydrophobic, meaning that they do not allow the passage of liquids as long as the maximum liquid entry pressure (LEP) is not exceeded [22]. The conditions chosen for the experiments ensured operation at a pressure below the LEP at all times. However, in some cases, liquid penetrates into the pores of membranes, causing pore wetting [22]. MD’s commercialization is hindered largely due to the occurrence of the pore-wetting phenomenon, since it results in a reduction in flux and/or permeate quality [5]. To evaluate the potential membrane deterioration and the occurrence of pore wetting, experiments were carried out using demineralized water, to which marine salts were added at a concentration similar to seawater (35 g/L), in addition to 25 µg/L of the selected contaminant, ketoprofen. Experiments with the same membrane were carried out over 108 h: 60 h at a hot-side temperature of 60 °C and 48 h at 80 °C. Samples of the feed, concentrate, and permeate were collected hourly. In MD processes, the temperature is a main factor affecting the permeate flux, since the driving force is the partial vapor pressure difference on both sides of the membrane generated as a consequence of a temperature difference established between both sides of the membrane [22,23]. During the presented investigations, at a lower temperature, an average volume of 347 ± 1.37 mL was produced during the first 22 h (permeate flux of 26.5 L/h·m^2^), followed by a 7% decrease thereafter (permeate flux of 24.7 L/h·m^2^) (Figure 3). However, this slight decline was not significant, and the permeate’s quality remained excellent (below 2 µS/cm) (Figure 4), with no detectable presence of ketoprofen [24]. The occasional spikes in higher conductivity (always lower than 6 µS/cm) are due to operational shutdowns. When operations resume, the volume of saline water that has dried inside causes an initial increase in conductivity. This effect is short-lived, as the production of distilled water quickly dilutes it and cleans out the remaining salt residues (Figure 4). At higher temperatures, the permeate’s volume during the initial 5 h averaged 667 ± 12 mL per hour (permeate flux of 50.9 L/h·m^2^), and it subsequently stabilized at around 620 ± 4 mL (permeate flux of 47.3 L/h·m^2^) (Figure 5). Once again, the permeate’s quality was excellent, with conductivity consistently below 2 µS/cm (Figure 6) and no traces of ketoprofen detected [24]. These results showed no deterioration of the MD membrane after 108 h of operation, i.e., the salts were rejected by the membrane, and no wetting was observed [24]. Sousa Mesquita et al. [25] attributed such phenomena to the higher hydrophobicity of PTFE membranes, which are characterized by superior stability and separation quality, especially under high-salinity conditions (7 g/L to 70 g/L) [25]. Similar values of ketoprofen removal (92–98%) were achieved by Woldemariam et al. [26] during purification of effluents from a municipal wastewater treatment plant via AGMD at a pilot plant. The highest permeate flux was achieved at a feed temperature of 90 °C and a cooling temperature of 15 °C [26]. The AGMD process was also applied for another type of organic contaminant, namely azo dyes: CI Reactive Red 241 (RR) and CI Acid Yellow 79 (AY) [27]. The oxygen plasma-modified PTFE membrane exhibited an increase in surface roughness and a water contact angle of 145°. The highest permeate flux for the modified membrane was 9.53 kg/m^2^·h, and a decrease with increasing dye concentrations was noted [27]. The observed results were explained by the gradual wetting of the membrane’s surface and its chemical interaction with dyes.

The characterization of the membrane’s surface after the process is essential in determining its efficiency, which is crucial for maintaining product quality. Post-membrane characterization provides information about the structural integrity and durability of the membrane. Moreover, fouling and scaling factors could be identified, enabling the development of strategies to minimize such phenomena [28].

**Figure 7:**
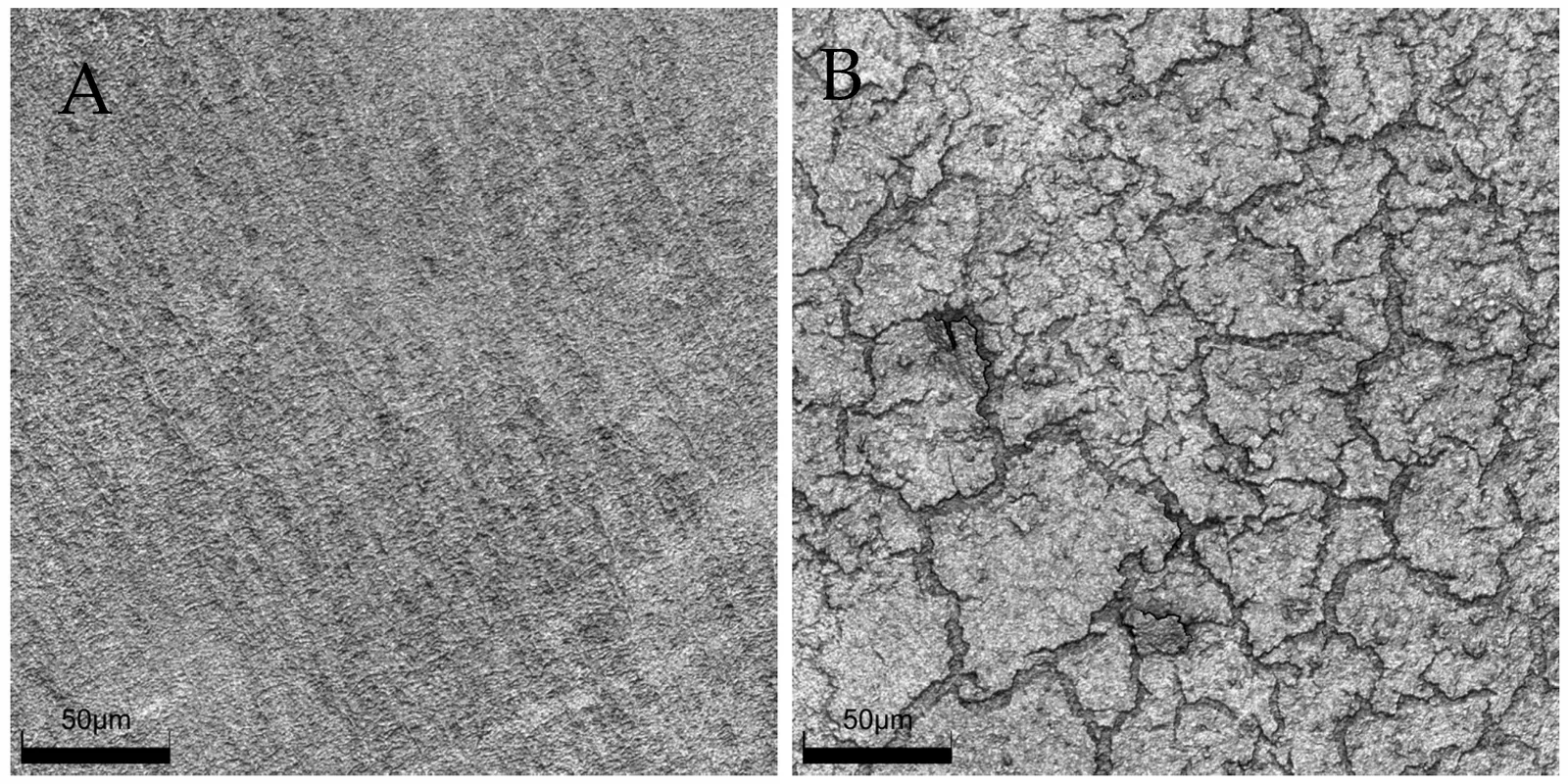
LSM microphotographs of the brand-new (**A**) and previous membrane after the AGMD process (**B**).

Analyses of the membrane surface (Figure 7) revealed that the membrane surface’s roughness, calculated based on the LSM, showed the formation of some deposits of salts [24]. Their presence resulted in a small increase in surface roughness due to exposure to seawater [16,28]. The S_a_ parameter was 0.268 µm and 0.397 µm for the brand-new and older membranes, respectively, and the S_q_ parameter was 0.336 µm and 0.519 µm after the experiment, respectively. The values of S_a_ and S_q_ indicated the formation of a very smooth layer during the MD process, which also confirmed that no wetting occurred on the membrane and that it exhibited high performance (Figure 5). The presence of deposits had no negative influence on distillate flux and quality, which shows the advantage of AGMD compared to systems utilizing pressure-driven membrane techniques [5,16]. The membrane surface coated with a thin layer of PTFE exhibited greater resistance to wetting compared to uncoated ones in the temperature range of 60–80 °C, thus maintaining stable performance over extended operation periods [29,30].

**Figure 8:**
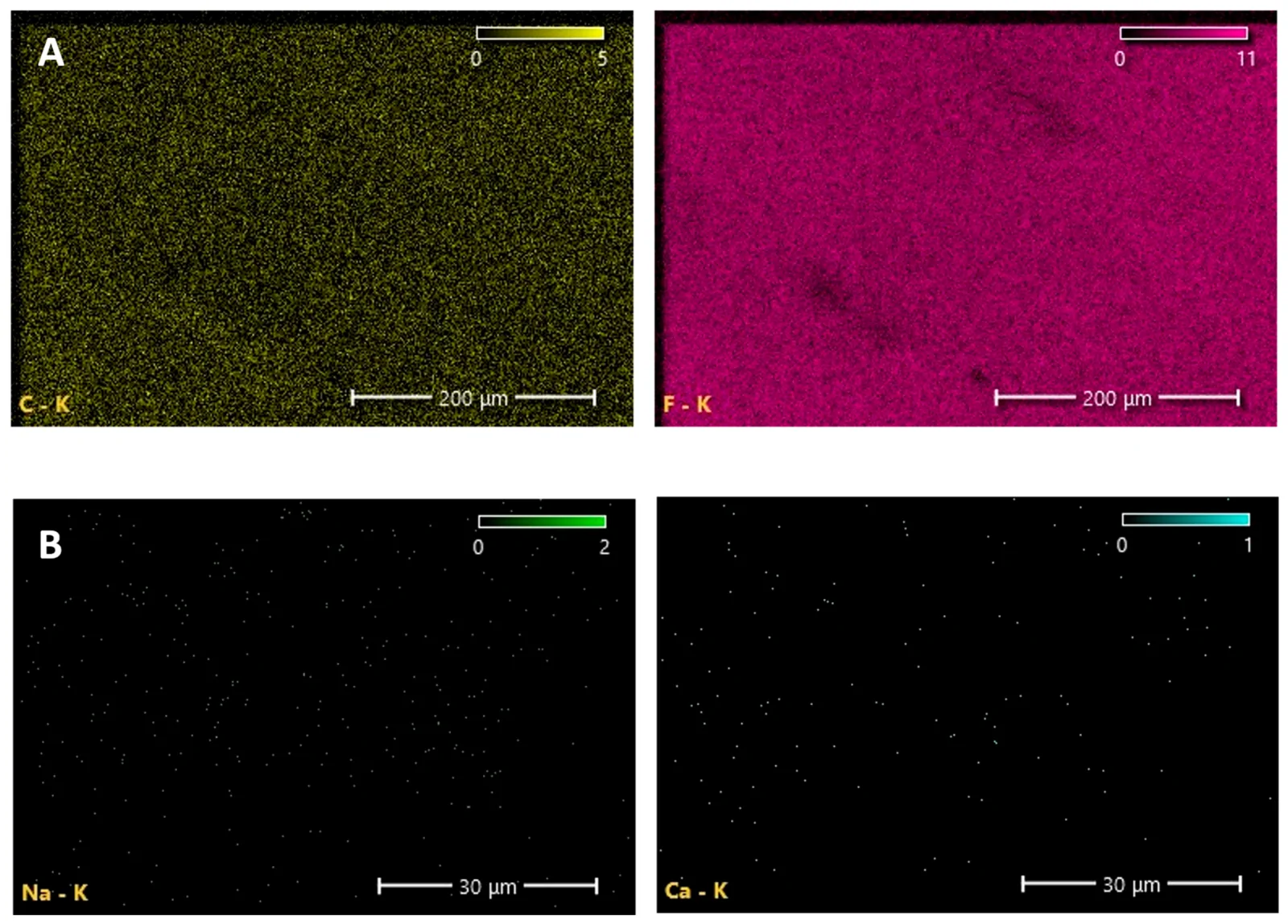
SEM-EDS images of the surface of the pristine membrane (**A**) and the membrane after the AGMD process (**B**). Color legend: yellow—C; pink—F; green—Na; blue—Ca.

SEM-EDS analyses revealed that the deposits formed on the membrane’s surface were mainly composed of Na and Ca (Figure 8B). It is worth noting that only trace amounts of these elements were detected on the membrane after the AGMD process. The presence of C and F, in turn, originated from the membrane material itself (PTFE) (Figure 8A).

### 3.2. Removal of Ketoprofen Under Photocatalytic Conditions

Numerous articles have investigated the effect of MD for the removal of endocrine-disrupting compounds (including pharmaceuticals) [26,31,32,33,34]. The data demonstrate that MD exhibits different retention efficiencies for these substances, ranging from ca. 60 to even 99.9%. Although the removal of EDCs from (waste)water by MD is an established phenomenon, resulting in pure distillates, which are products of the MD process, pollutant concentrations at the feed (retentate) side occur [35]. The concentrated EDCs in the feed solution still pose significant environmental risks if discharged without treatment. When further degradation treatment is carried out, these risks will be significantly reduced [35]. Therefore, there is an urgent need for concentrate management, e.g., via advanced oxidation processes [31,35]. Recent strategies involve membrane modification in terms of the photocatalysis process [36,37]; however, none of them take into account long-term stability under harsh photocatalytic operational conditions caused by hydroxyl radicals formed during the photocatalysis process. Such factors could damage polymeric MD membranes [14,15,38]. Another approach to solve this drawback is to separate the photocatalysis and MD process [12,16,35,39].

Since there were no traces of ketoprofen detected in the distillate from the AGMD process, the feed at this stage of the research was the concentrate from the AGMD experiment collected after 73 h.

**Figure 9:**
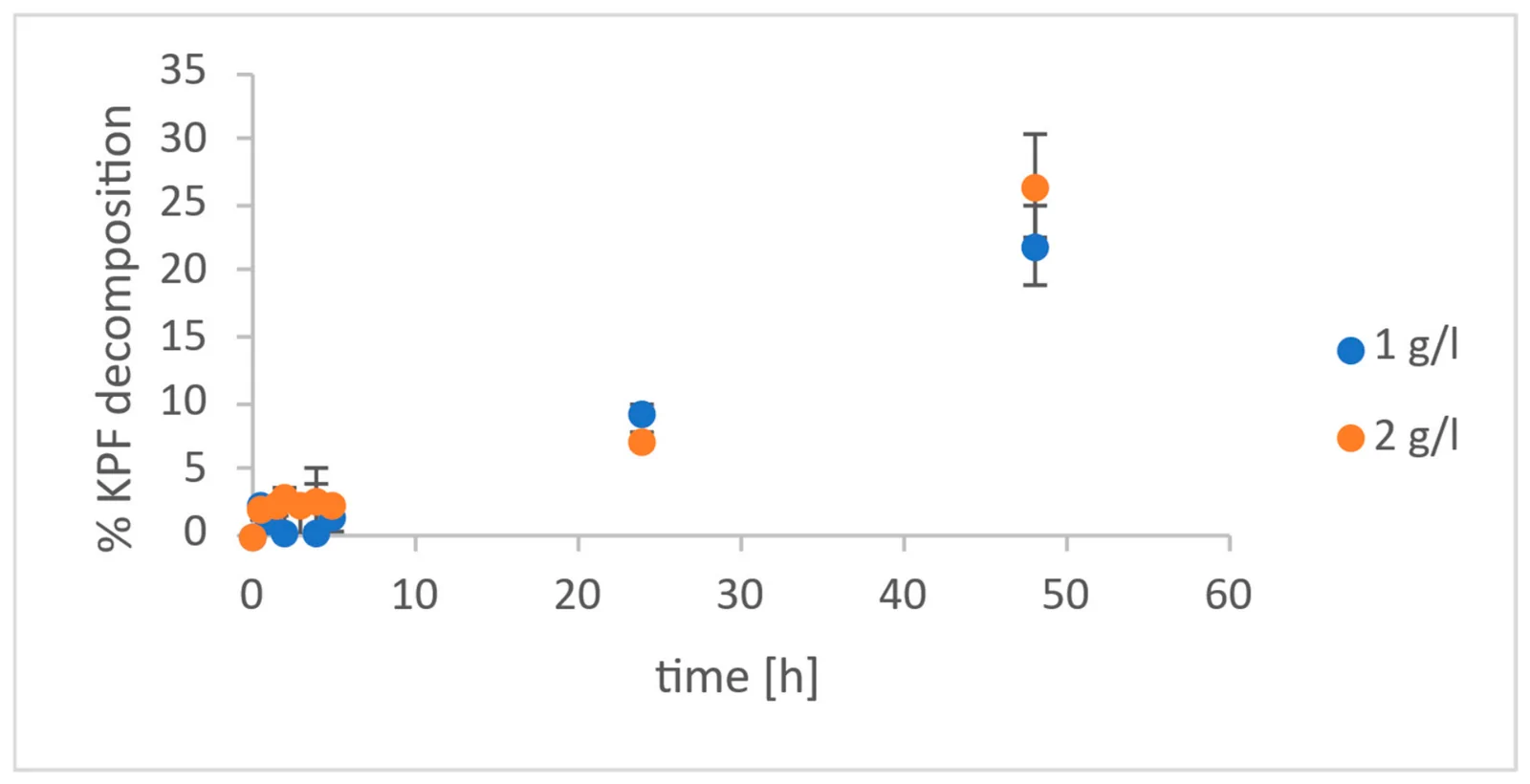
Ketoprofen removal during short-term photocatalysis process for various photocatalyst loadings.

In Figure 9, the influence of photocatalyst loading on ketoprofen removal under simulated sunlight is presented. It was found that after 48 h, ketoprofen removal still proceeded, and between loadings of 1 and 2 g/L of the photocatalyst, the difference in photodecomposition was negligible; therefore, for further experiments, 1 g/L of the P600 photocatalyst was used.

**Figure 10:**
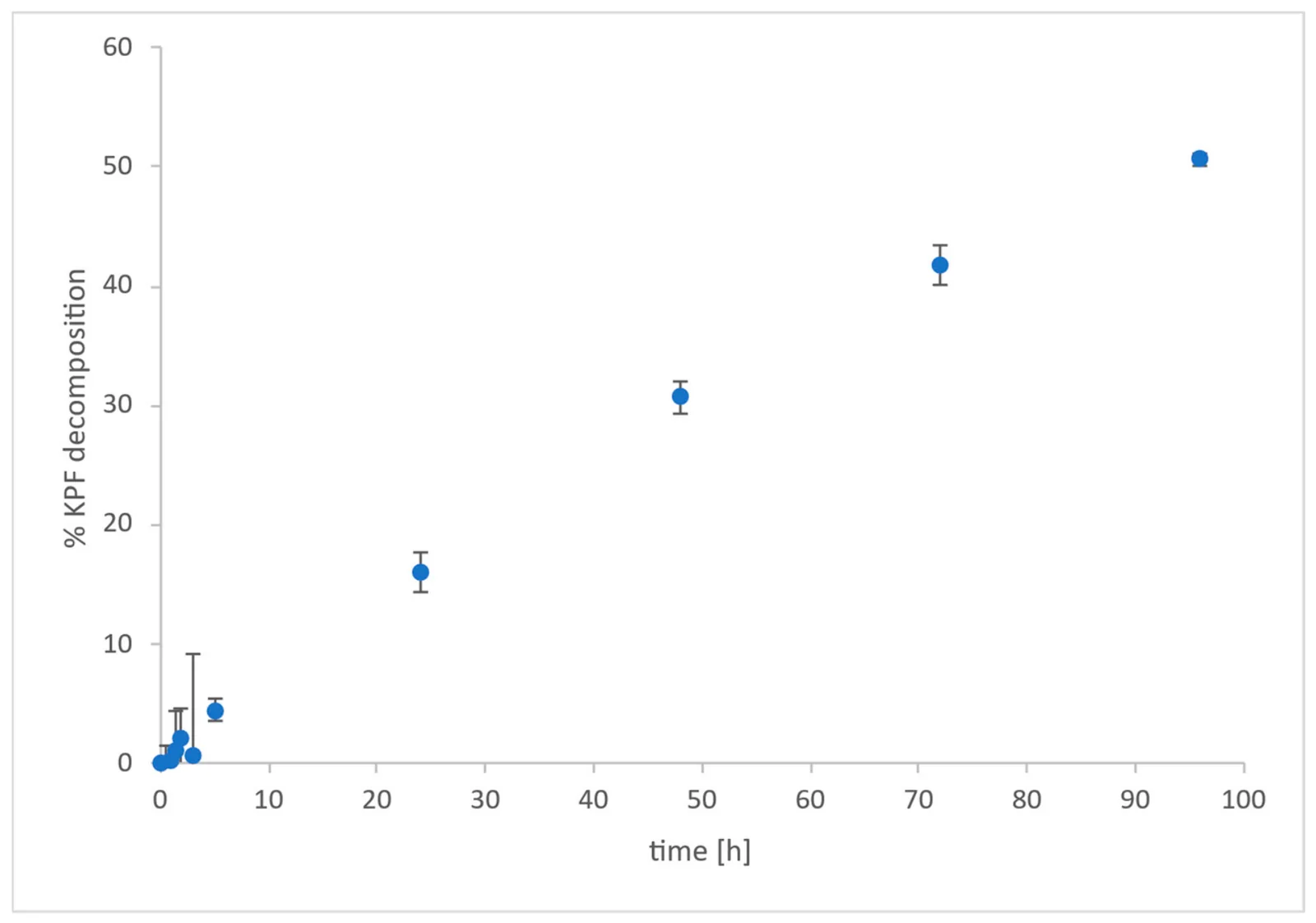
Ketoprofen removal during long-term photocatalysis process.

Figure 10 presents the results of four independent experiments investigating the photodecomposition of ketoprofen over a period of 96 h under simulated sunlight irradiation. The P600 photocatalyst used in the experiments showed high activity in sunlight [12,21,35]. The obtained results depended primarily on the excitation mechanism of the photocatalyst used in the experiments [18,40], and •O_2_^−^ radicals were the dominant reactive species in decomposition [14]. During the process, the pharmaceutical concentration systematically decreased, which was caused by photodecomposition via generated radicals (OH·) [35], reaching 51% removal after 96 h of the experiment. Although the removal degree was high, the feed water exhibited high salinity, and salts could act as scavengers [35,41]. The 51% ketoprofen removal achieved after 96 h of irradiation is lower than the values reported for visible-light photocatalysts based on Bi and reduced graphene oxide (rGO), for which ketoprofen degradation efficiencies ranging from 70% to 99% were obtained within 120 min to a few hours [42]. However, the obtained efficiency was higher than that reported for TiO_2_-based photocatalysis, where only 21% ketoprofen removal was achieved after 300 min of irradiation [43]. The lower efficiency observed in the present study may be attributed to the exceptionally high salinity of the treated matrix, which promotes radical scavenging and reduces photocatalytic activity. Furthermore, the superior performance of Bi/rGO-based photocatalysts has been attributed to the formation of a Schottky barrier and the rapid transfer of photogenerated electrons through rGO, which enhances charge separation and suppresses electron-hole recombination [42]. The lower performance observed in this work, despite the substantially longer irradiation time, suggests that matrix composition, particularly high salinity, plays a dominant role in limiting photocatalytic degradation. Chloride and other dissolved ions may compete with ketoprofen for reactive oxygen species, thereby decreasing the effective concentration of oxidizing radicals available for pollutant degradation. The degradation kinetics of ketoprofen in the high-salinity matrix were analyzed using the pseudo-first-order Langmuir–Hinshelwood model [42]. Based on the experimental data, the apparent kinetic rate constant was estimated as k = 7.4 × 10^−3^ h^−1^. The obtained value confirms the relatively slow photocatalytic degradation of ketoprofen under high-salinity conditions, which is consistent with the observed removal efficiency of 51% after 96 h of operation [10,43]. Overall, the photodecomposition followed by photocatalysis demonstrated satisfactory performance, achieving ketoprofen degradation ratios of up to 51% under the highest concentration of NaCl. These results are less promising compared with similar works, in which other organic compounds were photocatalytically decomposed; however, this should be emphasized in low-salinity water matrices [44,45,46]. For instance, Valadez-Renteria et al. [44] employed CuS/TiO_2_ nanoparticles in drinking water and obtained ~90% degradation of chlorophenol (20 mg L^−1^ initial concentration) after 150 min of irradiation [44]. Andrade et al. [47] obtained 70% phenol (85 ppm initial concentration) removal after 6 h of UV-visible light exposure using a composite of TiO_2_ and copper-loaded nanoporous carbon in a carbon matrix [47]. Vasantharaj et al. [45] observed similar results for TiO_2_ and ZnO particles in organic dyes (methylene blue and malachite green, 10 mg L^−1^ initial concentration). Researchers reached ca. 90% degradation after 150 min of sunlight irradiation in wastewater [45]. Lakshmi et al. [48] reported that La-ZnO-polyacrylonitrile fiber nanocomposites could achieve 98% degradation of atrazine (10 mg L^−1^ initial concentration), a persistent pesticide, in 60 min [48]. The observed degradation efficiency results from the combined effect of two factors: (i) relatively low photocatalytic activity of the P600 material under the applied irradiation conditions may have limited the generation of electron-hole pairs and, consequently, reduced the formation of reactive oxygen species responsible for ketoprofen degradation; (ii) the inhibitory effect of the saline matrix through radical scavenging and other competitive interactions.

The reaction between the organic contaminants and reactive radicals can lead to the oxidation of CO_2_ and H_2_O pollutants; however, this is more commonly observed with respect to other byproducts [35]. Solar-driven photocatalysis of ketoprofen led to the formation of byproducts, and two degradation pathways have been proposed. Three potential degradation intermediates were identified: a hydroxylated form of ethylbenzophenone, 3-acetylbenzophenone, and 3-acetylbenzophenone. The molecular masses of these intermediates were 211, 225, and 227 Da, respectively [42]. The identification of degradation intermediates is of great practical importance, since the oxidation of organic compounds may, in many cases, result in the formation of products that are more toxic than the parent pollutants. Such compounds may potentially pass through membranes and contaminate distillates. Nevertheless, in the presented research, conductivity during 96 h of the photocatalytic experiments was constant, which could indirectly suggest that intermediates of photodecomposition were mineralized [35]. Such MD–photocatalysis hybrid systems are characterized by the ability to retain non-volatile compounds and produce high-quality distillates [35]. Additionally, no traces of ketoprofen and salts were noted in the distillate.

Despite the fact that photocatalysis carried out under solar light is a very attractive direction of research due to applications of natural sunlight, thus minimizing treatment costs related to the energy needed to power lamps in the case of using UV irradiation, there are some limitations, such as the inability to operate the process at night and the need to develop sufficiently efficient photocatalysts that are active under sunlight and can compete with those operating solely under UV light [49,50,51]. Djouadi et al. [52] studied the photocatalytic decomposition of ketoprofen under UV–Vis irradiation using Bi_2_S_3_/TiO_2_-montmorillonite photocatalysts from water, with a very high efficiency reaching 80–100% depending on a pH value of 3 or 11; however, mineralization of organic compounds was only 16% after 2 h of treatment [52]. In turn, Sun et al. [53] reported very effective removal of ketoprofen (96% after 2 h) under visible light using MWCNT/N-TiO_2_/UiO-66-NH_2_ as a photocatalyst as well. The researchers attributed this high effectiveness to the improved visible-light absorption range, large specific surface area, and the formation of an effective heterojunction between the treated compound and photocatalyst [53].

## 4. Conclusions

During our research, a hybrid system combining air gap membrane distillation (AGMD) with solar-driven photocatalysis for the removal of ketoprofen from seawater was investigated. It was found that pure distillates free of salts (conductivity below 2 µS/cm) and ketoprofen were obtained after 73 h of operation.

The membrane’s performance showed approximately a twofold increase in permeate flux at an operating temperature of 80 °C compared to 60 °C, reaching 47.3 L·h^−1^·m^−2^ and 24.7 L·h^−1^·m^−2^, respectively. Despite the formation of small deposits on the membrane’s surface, no membrane wetting was observed.

The concentration of ketoprofen in the concentrate increased during the AGMD process; however, solar-driven photocatalysis was able to remove this pharmaceutical even from matrices enriched with NaCl (i.e., high-salinity AGMD concentrate), with good efficiency (51% after 96 h).

The proposed approach enabled the effective separation of ketoprofen from seawater while providing protection of the membrane against scaling and wetting. Furthermore, the photocatalytic treatment performed downstream of the membrane distillation (MD) process prevented the transfer of volatile organic compounds, generated as degradation intermediates, into the distillate. Additionally, the photocatalytic treatment of the MD concentrate intensified the degradation of ketoprofen, resulting in enhanced overall photodegradation efficiency.

The main challenge for further research is to identify a photocatalyst with higher activity under solar irradiation, which could enhance ketoprofen removal efficiency.

## Data Availability

The raw data supporting the conclusions of this article will be made available by the authors on request.

## Abbreviations

The following abbreviations are used in this manuscript:

AGMD	Air gap membrane distillation;
AOPs	Advanced oxidation processes;
C_0_	Initial concentration;
CECs	Contaminants of emerging concern;
CPCs	Compound parabolic collectors;
DCMD	Direct contact membrane distillation;
EDCs	Endocrine-disrupting chemicals;
FO	Forward osmosis;
HPLC	High-performance liquid chromatograph;
LEP	Liquid entry pressure;
LSM	Laser scanning microscopy;
MD	Membrane distillation;
MF	Microfiltration;
MWTP	Municipal wastewater treatment plant;
NF	Nanofiltration;
PMR	Photocatalytic membrane reactor;
PTCs	Parabolic trough collectors;
PTFE	Polytetrafluoroethylene;
rGO	Reduced graphene oxide;
RO	Reverse osmosis;
SPMR	Submerged photocatalytic membrane reactor;
TDSs	Total dissolved solids;
UF	Ultrafiltration;
UV	Ultraviolet;
WWTPs	Wastewater treatment plants.
